# Constitutive Expression of TERT Enhances β-Klotho Expression and Improves Age-Related Deterioration in Early Bovine Embryos

**DOI:** 10.3390/ijms22105327

**Published:** 2021-05-18

**Authors:** Lianguang Xu, Muhammad Idrees, Myeong-Don Joo, Tabinda Sidrat, Yiran Wei, Seok-Hwan Song, Kyeong-Lim Lee, Il-Keun Kong

**Affiliations:** 1 Division of Applied Life Science (BK21 Four), Gyeongsang National University, Jinju 52828, Gyeongnam Province, Korea; xulianguang428@gmail.com (L.X.); idrees1600@gmail.com (M.I.); jmd1441@gmail.com (M.-D.J.); tabindasidrat06@gmail.com (T.S.); weiyiran1230@gmail.com (Y.W.); 2 Institute of Agriculture and Life Science, Gyeongsang National University, Jinju 52828, Gyeongnam Province, Korea; 3 The Kingkong Co., Ltd., Gyeongsang National University, Jinju 52828, Gyeongnam Province, Korea; siwd2002@gmail.com (S.-H.S.); 0920-0728@hanmail.net (K.-L.L.)

**Keywords:** β-Klotho, FGFR1, TERT, cycloastragenol, bovine embryo

## Abstract

Age-associated decline in oocyte quality is one of the dominant factors of low fertility. Aging alters several key processes, such as telomere lengthening, cell senescence, and cellular longevity of granulosa cells surrounding oocyte. To investigate the age-dependent molecular changes, we examined the expression, localization, and correlation of telomerase reverse transcriptase (*TERT*) and β-Klotho (*KLB*) in bovine granulosa cells, oocytes, and early embryos during the aging process. Herein, cumulus-oocyte complexes (COCs) obtained from aged cows (>120 months) via ovum pick-up (OPU) showed reduced expression of β-Klotho and its co-receptor fibroblast growth factor receptor 1 (FGFR1). *TERT* plasmid injection into pronuclear zygotes not only markedly enhanced day-8 blastocysts’ development competence (39.1 ± 0.8%) compared to the control (31.1 ± 0.5%) and D-galactose (17.9 ± 1.0%) treatment groups but also enhanced *KLB* and *FGFR1* expression. In addition, plasmid-injected zygotes displayed a considerable enhancement in blastocyst quality and implantation potential. Cycloastragenol (CAG), an extract of saponins, stimulates telomerase enzymes and enhances *KLB* expression and alleviates age-related deterioration in cultured primary bovine granulosa cells. In conclusion, telomerase activation or constitutive expression will increase *KLB* expression and activate the FGFR1/β-Klotho pathway in bovine granulosa cells and early embryos, inhibiting age-related malfunctioning.

## 1. Introduction

The female reproductive system is more vulnerable to aging than other parts of the body, due to which women older than 35 years typically exhibit low fertility [1]. During the reproductive life, granulosa cell proliferation and secretory activities are required by the primary follicle to form an antral follicle [2]. Aging reduces the quality and quantity of granulosa cells, and as a result, the microenvironment (follicular fluid) of the oocyte deteriorates, which declines the oocyte quality [3,4]. Most aged oocytes show decreased fertilization rate, polyspermy, parthenogenesis, structural alteration, and hardening of the zona pellucida [5]. Furthermore, several intra-oocyte molecular processes are also affected by aging, such as chromosomal abnormalities, increased susceptibility to activating stimuli, decrease in maturation promoting factor (MEF), and mitogen-activated protein kinase (MAPK) [5]. Thus, knowledge of molecular biology is necessary to understand age-related malfunctioning in oocytes, their surrounding granulosa cells, and early embryos.

Increasing evidence demonstrates the age-associated decline in bovine fertility, and several molecular mechanisms have been explored to understand the process of aging in oocytes and early embryos [6,7]. Several anti-aging genes were identified that counteract the aging process and improve life span and fertility. β-Klotho (*KLB*) and telomerase reverse transcriptase (*TERT*) are anti-aging genes, and reduction in their expression enhances age-related malfunctioning in different tissues [8,9,10]. β-Klotho is a membrane-bound or soluble protein whose protective activity is essential for the proper functioning of many organs. Several previous studies have shown that *KLB* expression levels decrease with age and are associated with an increased risk of age-related diseases [11,12,13]. In mice, the overexpression of the *KL* gene increases the lifespan, whereas mutations in this gene reduce longevity [14]. *KLB* regulates life extension and derives much of its antiaging capacity by regulating telomerase (*TERT*) activity [15,16]. Klotho deficiency also accelerates stem cell aging by impairing TERT activity and inducing premature aging [17,18]. Telomerase is a ribonucleoprotein complex that adds hexameric (TTAGGG) telomeric DNA repeat sequences to the ends of eukaryotic chromosomes [19,20]. Telomerase is highly active in granulosa cells and differed according to follicle size and their localization in the follicles [21]. Granulosa cells are somatic cells but show high *TERT* expression instead of rapid division during follicle growth because granulosa cells can maintain their telomeric length [22,23]. Granulosa cells from old cows were found to have low telomerase activity and high reactive oxygen species (ROS) levels [24]. However, the specific molecular mechanisms responsible for β-Klotho regulated life extension via the modulation of telomerase activity are not well characterized, particularly within aging granulosa cells, oocytes, and early embryos.

In this study, we investigated the link between telomerase and β-Klotho and its co-receptor, FGFR1, in bovine oocytes, granulosa cells, and early embryos. To date, no studies have directly addressed the relation between telomerase and β-Klotho their anti-aging effects in bovine granulosa cells, oocytes, and early embryos. We first identified the expression levels of *KLB* and its co-receptor *FGFR1* in bovine cumulus-oocyte complexes (COCs) obtained from 5-years, 8-years, and 12-year-old cows. In 8 and 12-year old cows, the protein levels of β-Klotho and FGFR1 was lower than in 5-year-old cows. Furthermore, as early embryonic development progressed, β-Klotho and FGFR1 expression progressively increased in embryos. D-galactose treatment also reduces the expression levels of *KLB* and *FGFR1* in early bovine embryos and granulosa cells. *TERT* (pCI-neo-hTERT) plasmid injection into single-cell bovine zygotes or treating cultured primary bovine granulosa cells with cycloastragenol (specific activator of Telomerase) significantly enhances *KLB* gene expression. Also, it inhibits age-related malfunctioning in granulosa cells and early bovine embryos.

## 2. Results

### 2.1. Expression of β-Klotho in Bovine Oocyte and Early Embryos

**Figure 1:**
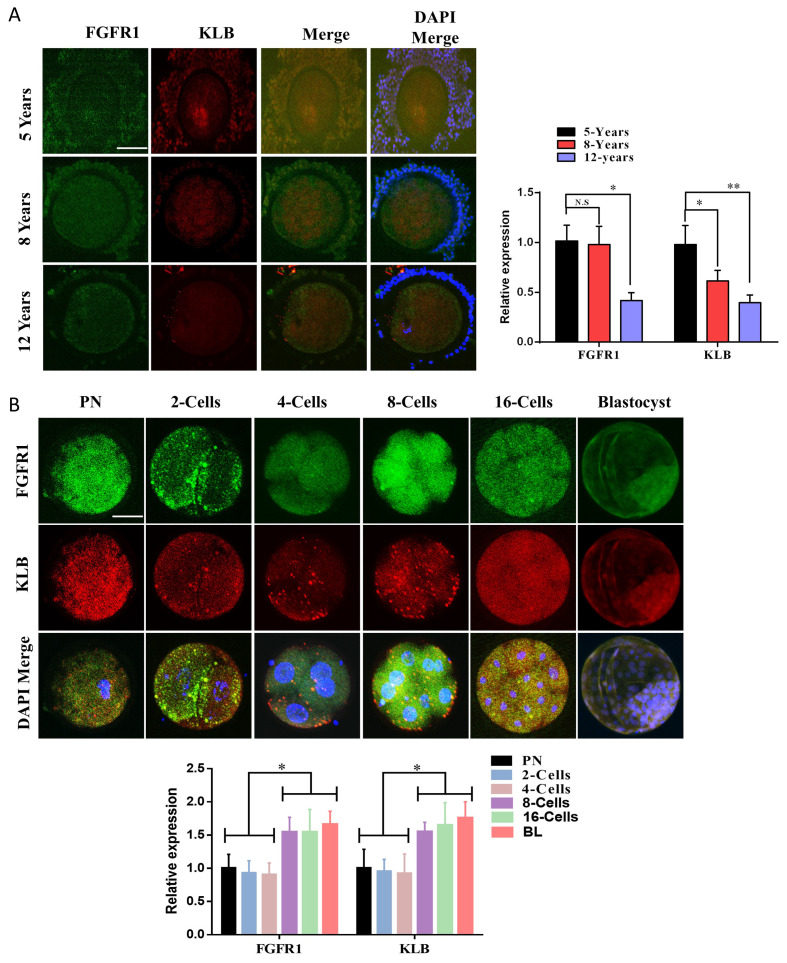
β-Klotho and FGFR1 proteins in bovine oocyte and early embryos. (**A**) The immunofluorescence based FGFR1 and β-Klotho proteins from Pronuclear (PN) stage to day-8 bovine blastocyst 10 per each sample), MII oocyte (20 per each sample), 2-cell embryo (20 per each sample), the 3.5-day embryo (10 per each sample) and day-8 blastocyst stages (5 per each sample and Bar = 100 µm). (**B**) Immunofluorescence expressions of FGFR1 and β-Klotho in bovine cumulus-oocyte complexes obtained from 5 years, 8 years, and 12 years old Hanwoo (Korea native) cows. The experiments were repeated 3 times, and the data are shown here as a mean ± S.E.M. N.S, not significant. Significance: * = *p* < 0.05, and ** = *p* < 0.01 and Bar = 100 µm.

To identify the relationship between KLB and aging in bovine embryos, we administered antibodies specific for β-Klotho and its co-receptor, FGFR1, to cumulus-oocyte complexes obtained from 5-to 12 years old Hanwoo cows via ovum pick-up (OPU) (Figure 1A). Based on immunofluorescence, there was a gradual decrease in β-Klotho protein expression with increasing age. Furthermore, in oocytes surrounding cumulus cells, the expression of β-Klotho was nuclear-localized, but an increase in age also reduced its nuclear localization. FGFR1 showed non-significant differential expression with increasing age in oocytes as well as cumulus cells. Additionally, the protein level of FGFR1 and β-Klotho from the pronuclear zygote (PN) stage to the day-8 bovine blastocyst (BL) stage were analyzed (Figure 1B). As development progressed, the protein levels of FGFR1 and β-Klotho were found to increase.

### 2.2. Aging Reduces the Expression of β-Klotho and Telomerase in Cultured Granulosa Cells

**Figure 2:**
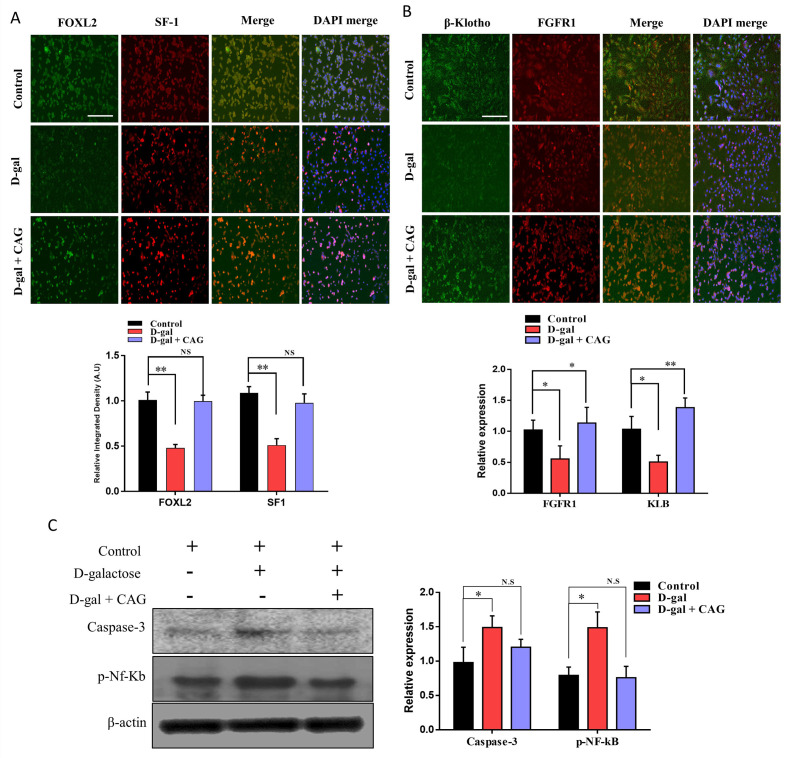
Aging reduces the expression of β-Klotho, and telomerase in cultured cumulus cells. (**A**) Immunofluorescence images of FOXL2 and SF-1 in bovine cultured granulosa cells treated with control, D-galactose, and D-galactose + cycloastragenol (the experiment was repeated three times independently and Bar = 100 µm). (**B**) Immunofluorescence expressions of β-Klotho and FGFR1 show significant (*p* > 0.05) reduction with D-galactose treatment (Bar = 100 µm). (**C**) Western blot expression of Caspase-3 AND NF-Kb in cultured bovine granulosa cells treated with control, D-galactose, and D-galactose + cycloastragenol. The experiment was repeated three times and the data are shown here as a mean ± S.E.M. N.S, not significant, * = *p* < 0.05, and ** = *p* < 0.01.

Oocytes and the surrounding granulosa communicate with each other to achieve successful maturation and fertilization. Granulosa cells’ ability to proliferate around the oocyte and provide nutrients to the oocyte decreases with maternal age. Several differentially expressed genes have also been identified in aged granulosa cells [3]. We cultured bovine granulosa (cumulus) cells and treated them with d-galactose and d-galactose + cycloastragenol (CAG) (Thereafter, FOXL2 (granulosa cell-specific marker) and steroidogenic factor-1 (SF-1) were analyzed by immunofluorescence (Figure 2A). Based on our findings, there was no significant difference between FOXL2 and SF-1 expression in the control, d-gal, and d-gal + CAG groups. We proceeded to verify the expression of FGFR1 and β-Klotho. We found that both proteins showed a significant reduction in expression after treatment with D-galactose, while CAG neutralized the D-galactose effect (Figure 2B). By analyzing the expression of caspase-3 and NF-kB, we found that d-galactose treatment enhanced apoptosis of cultured granulosa cells, but the TERT stimulator, CAG, significantly (*p* < 0.05) neutralized this effect.

### 2.3. TERT Plasmid Injection Enhances KLB Protein Expression and Inhibits ROS Level

**Figure 3:**
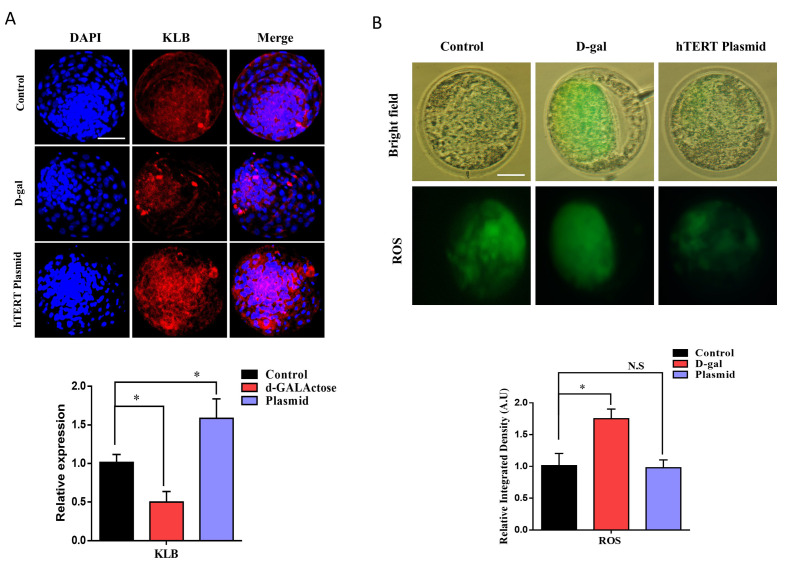
TERT plasmid injection enhances KLB protein expression and inhibits ROS level. (**A**) Immunofluorescent expression of KLB in bovine day-8 blastocysts treated with control, D-galactose, and TERT plasmid. (**B**) Reactive oxygen species (ROS) level was measured via H2DCFA staining in bovine day-8 blastocysts. The result showed a significant reduction in ROS level with hTERT plasmid injection. The data shown here as a mean ± S.E.M. N.S, not significant, * = *p* < 0.05, and Bar = 100 µm.

A previous study revealed that Klotho protein level is directly proportional to telomerase activity. To identify the link between telomerase and β-Klotho, we injected TERT plasmid into bovine pronuclear (PN) zygotes and cultured them in IVC medium for 8 days (Table 1). Thereafter, we analyzed β-Klotho expression and compared it to the control and D-galactose-treated day-8 blastocysts (Figure 3A). From the results, we identified that the TERT plasmid significantly enhanced β-Klotho expression compared to the control, while D-galactose reduced its expression. We also examined the level of ROS, which was also significantly (*p* < 0.05) reduced in the day-8 bovine embryos injected with the TERT plasmid (Figure 3B). The above results suggest that increased telomerase activity improves embryo quality and negatively affects aging-related malfunctioning during early embryonic development.

### 2.4. Enhanced TERT Expression Amplifies Mitochondrial Genes and Improves Mitochondrial Potential

**Figure 4:**
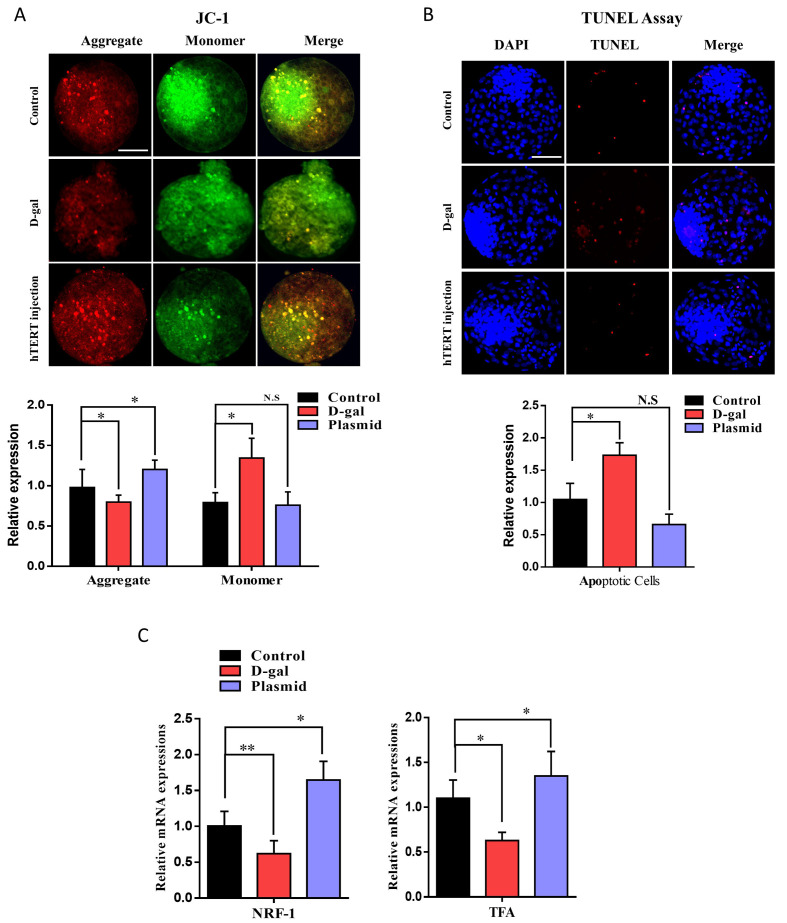
Enhanced telomerase expression amplifies mitochondrial genes and improves mitochondrial potential. (**A**) JC-1 staining in day-8 blastocysts from the control, D-galactose, and hTERT-treated groups (20 per each group) and Bar = 100 µm. (**B**) TUNEL assay was performed to detect apoptotic cells. The D-galactose group had significantly more TUNEL positive cells than the control and TERT plasmid injected groups (*n* = 20 per group) and Bar = 100 µm. (**C**) The relative mRNA expressions of NRF-1 and TFA in bovine day-8 blastocysts. The expression of both the genes were significantly reduced in D-gal group as compared to control and Plasmid groups. The data shown here as a mean ± S.E.M. N.S, not significant, * = *p* < 0.05, and ** = *p* < 0.01.

The mitochondria play a vital role in early embryonic development, and maternal age markedly affects the mitochondrial copy number and ATP content in early embryos [25]. To analyze the effects of the TERT plasmid on the active mitochondrial membrane potential, we carried out JC-1 staining. JC-1 staining revealed that the TERT plasmid significantly (*p* < 0.05) enhanced active mitochondria, whereas d-galactose had negative effects on mitochondrial aggregates (Figure 4A). We also investigated apoptotic cells via TUNEL assay on day 8 bovine blastocysts, which revealed significantly low apoptosis in TERT plasmid-injected embryos than the control and d-gal counterparts (Figure 4B). Furthermore, *NRF-1* and *TFA* mRNA levels were analyzed by RT-qPCR. Based on the results, both the genes were significantly reduced with d-galactose; however, their expression levels were significantly enhanced by the TERT plasmid (Figure 4C). The above results indicate that TERT-enhanced expression positively affects embryo mitochondrial functioning and inhibits apoptotic signals.

### 2.5. Constitutive Expression of TERT Enhances Embryo Implantation Potential and Inner Cell Mass (ICM)-Related Genes

**Figure 5:**
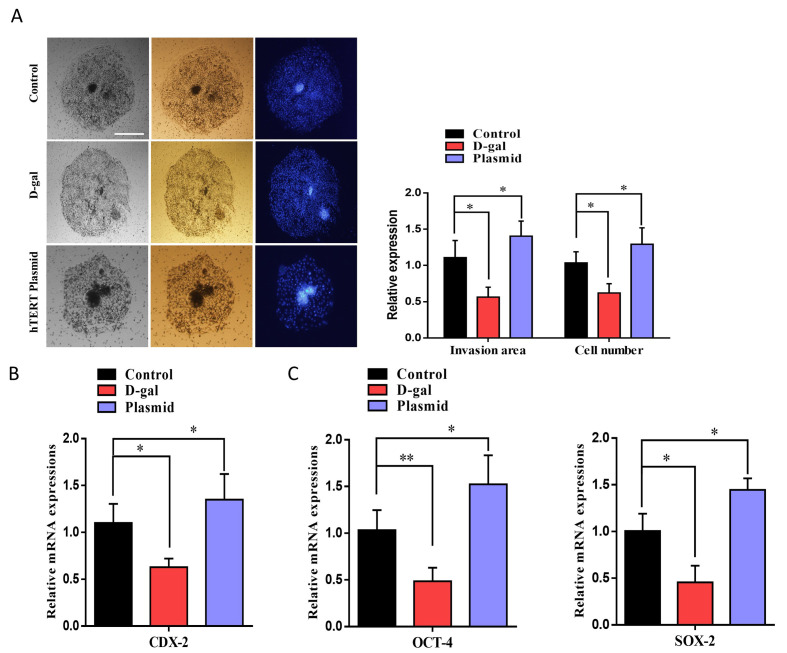
Constitutive expression of TERT enhances embryo implantation potential and inner cell mass (ICM)-related genes. (**A**) The effects of D-galactose and TERT on blastocyst implantation potential were determined via invasion assay. Bright-field image showing the area of invasion and DAPI for migrant cells in day-8 blastocysts. Image J software (National Institute of Health, Bethesda, MD, USA) was used to quantify the signal intensity of immunofluorescent images. The invaded area and cellular migration were significantly lower in the D-galactose-treated group than the control and TERT-treated groups and Bar = 100 µm.(**B**) CDX-2 gene related to embryo implantation and invasion of maternal uterine tissue was analyzed via RT-qPCR. (**C**) Inner Cell Mass (ICM) related genes like OCT-4 and SOX-2 were analyzed to check the embryo quality. The results showed that both the genes were significantly reduced with D-galactose treatment compared to control TERT plasmid injection. The experiments were repeated 3 times, and the data are shown here as the mean ± S.E.M. * = *p* < 0.05, and ** = *p* < 0.01.

To analyze the effect of TERT plasmid on embryo implantation potential and its invasion of maternal uterine tissue, we used an invasion assay [26]. The embryonic implantation potential of the TERT plasmid-injected group was significantly high as compared to the control and d-galactose-treated groups (Figure 5A). Furthermore, the area of invasion and cell number was also significantly higher in TERT plasmid embryos than the control and d-galactose-treated embryos. Moreover, *CDX-2*, which plays a critical role in embryo implantation and placenta formation, also showed enhanced expression by TERT plasmid injection (Figure 5B). After that, we examined the mRNA expression of ICM-related genes, such as *OCT-4* and *SOX-2*. Both the genes showed significant expression in hTERT plasmid-injected embryos compared to control d-galactose-treated embryos (Figure 5C). Collectively, the above results suggest that telomerase activation with cycloastragenol or TERT plasmid injection has significant effects on cultured bovine granulosa cells and early bovine embryos.

## 3. Discussion

Currently, limited information is available regarding age-dependent changes in bovine granulosa cells, oocytes, and early embryos. Age-associated decline in oocyte quality and early embryo development is common in mammals [6,7]. Oocytes take a long time to reach their full size in large animals, and maternal physical conditions profoundly affect follicle development. In this study, we attempted to determine the association between two anti-aging related genes (*TERT* and *KLB*) and their differential expression in bovine granulosa cells, oocytes, and early embryos during aging. We found that β-Klotho and its co-receptor FGFR1 show reduced expression in aged Hanwoo cows (12 years) as compared to young (5 years) cows. D-Galactose treatment also significantly reduced β-Klotho expression in granulosa cells, oocytes, and early embryos. TERT plasmid injection or its activation by CAG significantly enhanced β-Klotho expression and neutralized D-galactose induced toxicity in granulosa cells and early embryos.

For decades, scientists have searched for genes that regulate lifespan. Klotho was discovered in a transgenic mouse strain in 1997, and its mutation caused a syndrome similar to premature aging, including reduced lifespan, growth retardation, vascular calcification, genital atrophy, emphysema, and osteomalacia [16,27]. *KL* is an antiaging protein predominantly produced in the kidney, with the shedding of secreted Klotho (sKL) into the systemic circulation [14,28]. In our study, β-Klotho (*KLB*) and its co-receptor were identified to be expressed in granulosa cells, germinal vesicle (GV) stage oocytes, and day-8 bovine blastocysts (Figure 1). Further, the expression levels of both proteins were significantly enhanced during the embryonic developmental stages. By analyzing the expression of β-Klotho and its co-receptor, FGFR1, in granulosa oocyte complexes (COCs) obtained from aged cows via OPU, we found that increasing age significantly reduced the expression of KLB. A previous study revealed that the hindered expression of Klotho impairs telomerase activity [17]. We activated TERT in cultured bovine granulosa cells using its activator, CAG, and found that telomerase activation significantly increased β-Klotho and FGFR1 in cultured bovine granulosa cells (Figure 2).

The germ cell develops inside the somatic cells (granulosa cells) of the ovary and matures to a fertilizable zygote during the reproductive life of mammalian females, a mechanism known as folliculogenesis. During folliculogenesis granulosa cells rapidly proliferate and divide throughout the subsequent follicular development [29]. In normal somatic cell cycle, telomeric DNA replication results in telomere length shortening due to end replication issues and undergo cell senescence [30]. Oocytes surrounding somatic cells have high telomerase activity; however, telomere length is markedly reduced in aging conditions. In fact, granulosa cells from old cows were found to have low genomic global DNA methylation, reduced proliferative and telomerase activity, and shortened telomeres [24]. The protein expression of GCs was reported to differ between young (20–33 years) and elderly (40–45 years) women. Further, 110 (7.7% in total) proteins were found to be differentially expressed in aging GCs. We found that D-galactose treatment significantly reduced the expression of surrogate markers, such as FOXL2 and SF-1, while TERT activation with CAG retained the expression of these proteins (Figure 2). β-Klotho and FGFR1 were also identified to be significantly affected by D-galactose treatment compared to the control. By analyzing the combined treatment of D-galactose and CAG, we found that CAG significantly neutralized d-galactose-induced malfunctioning in bovine granulosa cells. Recent studies have suggested that in addition to maintaining telomere length, telomerase is also involved in other cellular functions of biological relevance [31]. Herein, we found that cycloastragenol activation of telomerase inhibited the apoptotic pathways stimulated by D-galactose treatment in cultured bovine granulosa cells.

Aged oocytes have a reduced ability to counteract ROS, which is one of the main causes of cellular injury associated with aging. Furthermore, ROS is also one of the main factors in the nuclear export of telomerase, and it is also inversely proportional to β-Klotho [4,32]. We injected TERT plasmid into bovine zygotes and cultured it for 8 days to analyze ROS level and β-Klotho expression level. Based on our findings, TERT plasmid-injected blastocysts showed reduced ROS levels compared to the control and D-galactose-treated embryos (Figure 3). Furthermore, plasmid-treated blastocysts showed significant enhancement in *KLB* expression. Improvement in ROS levels indicates proper functioning of the mitochondria, which play a critical role in early embryo development [33]. We found that the mitochondrial membrane potential (ΔΨm) was significantly higher in TERT plasmid-injected embryos than D-galactose-treated embryos (Figure 4). The embryo implantation potential of the TERT plasmid-injected group was also analyzed and compared to that of the control and D-galactose-treated groups (Figure 5). Both the area of invasion and cell number was significantly higher in TERT plasmid embryos than control and D-galactose-treated embryos. Furthermore, the expression of *CDX-2* (involved in maternal uterine tissue invasion) was found to be enhanced by TERT plasmid injection [34]. Moreover, the mRNA expression levels of ICM-related genes, such as *OCT-4* and *SOX-2*, were also significantly higher in TERT plasmid-injected embryos than control and D-galactose-treated embryos. Altogether, these findings suggest that telomerase activation with cycloastragenol or TERT plasmid injection has significant effects on β-Klotho expression and the aging process in cultured bovine granulosa cells and early bovine embryos.

## 4. Materials and Methods

All experiments were conducted with slaughterhouse-derived materials. The Gyeongsang National University Institute of Animal Care Committee approved all experiments, including surgical procedures (GNU-130902-A0059, approved on 2 September 2013). All of the chemicals and reagents were obtained from Sigma-Aldrich (St. Louis, MO, USA), unless otherwise noted.

### 4.1. Aspiration of Oocyte and In Vitro Maturation (IVM)

Oocyte aspiration and in vitro maturation procedures were performed as described previously [35]. Briefly, ovaries collected from the abattoirs were washed with Dulbecco’s phosphate-buffered saline (D-PBS). The aspirated fluid was then dispensed into 100-mm Petri dishes containing Tyrode lactate-HEPES (TL-HEPES) medium. Oocytes with a compact layer of cumulus cells were collected and washed with TL-HEPES medium. Approximately 50 cumulus-oocyte complexes (COCs) were sequentially matured in 4-well plates (Nunc, Roskilde, Denmark) containing 700 µL of IVM medium consisting of TCM-199 (Invitrogen Corp., Carlsbad, CA, USA) supplemented with FBS (10%), oestradiol-17β (1 µg/mL), follicle-stimulating hormone (10 µg/mL), epidermal growth factor (10 ng/mL), cysteine (0.6 mM), and Na-pyruvate (0.2 mM) under 5% CO_2_ at 38.5 °C for 22–24 h.

### 4.2. In Vitro Fertilization (IVF) and In Vitro Culture of Embryos

For in vitro fertilization, the frozen bovine semen was thawed in a water bath at 37 °C for 1 min and washed with D-PBS via centrifugation at 1800 × rpm for 5 min at room temperature (RT). The pellet collected from the bottom of the tube was re-suspended in 500 µL of 20 µg/mL heparin prepared in IVF medium (Tyrode lactate solution supplemented with BSA (6 mg/mL), sodium pyruvate (22 mg/mL), penicillin (100 IU/mL), and streptomycin (0.1 mg/mL)], followed by incubation at 38.5 °C in a humidified atmosphere with 5% CO_2_ for 15 min to facilitate capacitation. Spermatozoa were diluted with IVF medium to a final concentration of 1 × 10^6^ cells/mL. Matured COCs were transferred to 4-well dishes containing sperm in 600 µL of IVF medium. These dishes were incubated in a humidified atmosphere with 5% CO_2_ at 38.5 °C for 18–20 h. After co-culture with spermatozoa for 20 h, oocytes were cleared from the cumulus cells by repeated pipetting. Thereafter, denuded presumptive zygotes were washed and transferred to four-well dishes containing 600 µL of SOF medium supplemented with BSA (4 mg/mL), insulin (5 mg/mL), transferrin (5 mg/mL), sodium selenite (5 ng/mL), and EGF (100 ng/mL).

### 4.3. Cytoplasmic Injection

After 5 h of culture in IVF medium, cumulus cells were stripped from COCs by repeated pipetting in 0.1% (*v/v*) bovine testicular hyaluronidase prepared in TL-HEPES. Denuded oocytes with first polar bodies were selected for cytoplasmic injection, as previously described [36]. The hTERT plasmid (pCI-Neo-hTERT) prepared in 10 mM Tris-HCl (pH 8.0) and 0.25 mM EDTA (pH 8.0), at a final concentration of 10 ng/μL and backfilled into microinjection capillaries. Individual zygotes were immobilized by suction to a holding pipette while the injection capillary was manually pushed through the zona pellucida and cell membrane. Approximately 10 pL of plasmid solution was injected into the zygote cytoplasm. The essential micromanipulation medium was TCM-199 supplemented with 0.3% BSA. After injection, the zygotes were washed twice in the culture medium and transferred into culture drops.

### 4.4. Primary Bovine Granulosa Cell Culture

Granulosa cells were obtained from 3–6 mm COCs and cultured according to previously described media and conditions [37,38]. Briefly, cumulus cells were obtained from COCs isolated via persistent pipetting with a narrow pipette. The collected cumulus cells were cultured on collagen-coated 24-well plates with 1.25 × 105 viable cells per well in a 6-well dish. Serum-free α-MEM medium was supplemented with HEPES (20 mM), BSA (0.1%), sodium bicarbonate (0.084%), sodium selenite (4 ng/mL), transferrin (5 μg/mL), insulin (10 ng/mL), androstenedione (2 μM), L-glutamine (2 mM), nonessential amino acids (1 mM), penicillin (100 IU), and streptomycin (0.1 mg/mL).

### 4.5. Reactive Oxygen Species Determination in Embryos

In the experiments, the ROS content of IVF, D-gal, and hTERT plasmid-injected embryos was measured at the blastocyst stage. The level of ROS content was quantified using 2′,7′-dichlorodihydrofluorescein diacetate (DCHFDA, fluorescent probe, D-6883), as previously described [26]. Fluorescence images were captured using a confocal laser microscope (Olympus, FV1000, Tokyo, Japan), and quantitation was carried out using Image J software (National Institutes of Health, Bethesda, MD, USA).

### 4.6. Immunofluorescence Analysis

Immunofluorescence staining was performed as described previously [38]. Briefly, samples were fixed with 4% (*w/v*) paraformaldehyde solution and permeabilized with 0.5% Triton X-100. After blocking, the sections were sequentially incubated with primary antibody overnight followed by secondary antibodies conjugated with FITC or TRITC at RT for 1 h. After the sections were washed extensively with PBS/polyvinylpyrrolidone (PVP), the nuclei were counterstained with DAPI at RT for 5 min. The samples were washed three times with PBS for 5 min, treated with 4,6-diamidino-2-phenylindole (DAPI) for 10 min to stain the nucleus, and fixed on slides. The slides containing the samples were covered with glass coverslips using a fluorescent mounting medium. Images were captured using a confocal laser-scanning microscope (Fluoview FV 1000; Olympus, Tokyo, Japan). To measure the relative integrated density, signal and area were obtained using the ImageJ software.

### 4.7. Immunoblotting

For protein extraction, the trypsinized cells were washed with D-PBS and dissolved in PRO-PREP™ (cat. 17081, iNtRON Biotechnology, Burlington, NJ, USA). Thereafter, the samples were sonicated, and the cell lysate was centrifuged at 10,000× *g* at 4 °C for 25 min [38]. The proteins in the supernatant were quantified using the Bradford assay (cat. 5000002 Hercules Laboratories, CA, USA), and the settled debris was discarded [37,38]. Equal amounts of protein (20 µg) were fractionated via SDS-PAGE (10% and 12%) and transferred onto a polyvinylidene fluoride (PVDF) membrane (sigma-Aldrich, St. Louis, MO, USA cat. # GE10600023). Skimmed milk was applied for 1 h to block the PVDF membrane. After blocking, the PVDF membrane was incubated overnight with a primary antibody at 4 °C in a two-dimensional shaker. The next day, the membrane was incubated at RT with an HRP-conjugated secondary antibody for 90 min. To detect the bound antibodies, an enhanced chemiluminescence (ECL) detection reagent (Pierce TM ECL western blotting Substrate, Thermo Fisher Scientific, Waltham, MA USA) was used. To determine the molecular weight of the proteins, protein ladders (cat. ab116029) were employed. ImageJ software (https://imagej.nih.gov/ij, accessed on 1 March 2021) was used to detect the optical densities of the bands on X-ray films (iNtRON Biotechnology, Inc., Burlington, NJ, USA).

### 4.8. TUNEL Assay

The TUNEL assay was performed according to the manufacturer’s protocol using an in situ cell death detection kit (Roche Diagnostics Corp., Indianapolis, IN, USA) [26]. Briefly, fixed embryos were washed with PBS/PVP and permeabilized in 0.5% (*v/v*) Triton X-100 and 0.1% (*w/v*) sodium citrate at RT for 30 min. Thereafter, the blastocysts were incubated in the dark with TUNEL solution at 37 °C for 1 h. Stained embryos were washed with PBS/PVP and incubated in DAPI for 10 min. After washing with PBS/PVP, the blastocysts were mounted on a glass slide, and their nuclear configurations were analyzed. The number of cells per blastocyst was determined by counting the DAPI-stained cells under an epifluorescence microscope (Olympus IX71) equipped with a mercury lamp. TUNEL-positive cells were labeled bright red (apoptotic) while normal cells were labeled blue.

### 4.9. JC-1 Staining

Mitochondrial ∆Ψ m in blastocysts was analyzed via staining with JC-1 (Molecular Probes, Invitrogen, Carlsbad, CA, USA) [39]. Briefly, day-8 blastocysts were collected and fixed in 4% paraformaldehyde, washed, and incubated with 10 mg/mL JC-1 dye prepared in PBS/PVP solution at 37 °C for 1 h under dark conditions. In principle, the dye is incorporated into the mitochondria and generates either a green fluorescence signal via monomer formation (J-monomer), indicating a low membrane potential, or a red fluorescence signal via aggregate formation (J-aggregate), indicating high membrane potential. The blastocysts were washed with PBS/PVP solution and stained with DAPI for 5 min. After washing, the blastocysts were mounted on a glass slide with a coverslip. Images were captured using a confocal laser scanning microscope (Fluoview FV 1000, Olympus, Tokyo, Japan).

### 4.10. Invasion Assay

To quantify invasion, Day-8 blastocysts were placed on an invasion chamber insert (6.4 mm; Corning Inc. Life Sciences Corning, New York, NY, USA) containing polyethylene terephthalate membranes (8 µm-diameter pores) in 24-well tissue culture plates (Corning Inc. Life Sciences Corning, New York, NY, USA). The upper surface of the chamber was coated with Matrigel (20 mg per filter; Discovery Labware Inc., Billerica, MA, USA) and incubated at 37 °C for 2 h to allow drying. The blastocysts were added to the filter coated with Matrigel (three blastocysts per culture insert suspended in the same medium used for embryo production) and incubated in a humidified atmosphere with 5% CO_2_ at 37 °C for 72 h. The medium was replaced with a fresh medium every 24 h at the bottom of the culture chamber. After 10 days of culture, the invasion and spreading area of trophoblasts were evaluated under a phase-contrast Olympus IX71 microscope and analyzed using Image J software. Subsequently, a cotton swab was used to scrub the upper surface insert membrane of the chamber. Cells were fixed with 4% (*w/v*) paraformaldehyde on the lower surface of the scrubbed membrane for 15 min at RT and stained with DAPI for 10 min. Cells transported across the membrane were counted under a phase-contrast Olympus IX71 microscope.

### 4.11. RNA Extraction and Quantitative Real-Time PCR (qRT-PCR) Analysis

Total RNA from day-8 blastocysts (*n* = 5 per group) was isolated using an RNA isolation kit (PicoPure, ThermoFisher, Arcturus, Foster, CA, USA). Subsequently, cDNA synthesis and quantitative real-time polymerase chain reaction (qRT-PCR) were performed using iScript Reverse transcriptase (Cat # 170889, BioRad,) and SYBR Green master mix (Cat # 170-8882AP, BioRad), respectively. The following PCR conditions were employed: 94 °C for 5 min, followed by 40 cycles of 94 °C for 30 s, 58 °C for 30 s, and 72 °C for 30 s. The mRNA expression levels were normalized to that of glyceraldehyde-3-phosphate dehydrogenase (GAPDH) and are expressed as the fold change. For the mRNA expression analysis, the experiments were performed in triplicate. The primers used for qRT-PCR are listed in Table 2.

### 4.12. Statistical Analysis

A computer-based Sigma Gel System (SPSS Software Inc., Chicago, IL, USA) was used for embryo development analysis. To analyze the density and integral optical density (IOD) of scanned X-ray films of Western blot and immunofluorescence images, Graph Pad Prism 6 (GraphPad Software, San Diego, CA, USA.) and Image J software (National Institute of Health, Bethesda, MD, USA) were used. To determine the statistical significance (*p*-value), one-way ANOVA followed by Student’s t-test was used to analyze the data. The density values of the data are expressed as the mean ± SEM of three independent experiments. Significance: * = *p* < 0.05, ** = *p* < 0.01, and *** = *p* < 0.001.

## 5. Conclusions

In this study, we attempted to determine the link between telomeraseand β-Klotho in bovine granulosa cells, oocytes, and early embryos. We found that the activation of telomeraseusing cycloastragenol (CAG) or TERT plasmid injection significantly enhanced β-Klotho expression. Furthermore, age-related malfunctioning in granulosa cells and early bovine embryos was found to be significantly reduced with telomerase activation. A dysfunction in the aging/ROS-FGFR1-β-Klotho-telomerasesignaling pathway may thus be involved in the aging of the oocyte and its surrounding somatic cells. Furthermore, telomeraseactivation or its enhanced expression can neutralize this mechanism of aging.

## Figures and Tables

**Table 1:** In Vitro Development of Embryos Cloned Using Different Methods.

Groups	No. of Cultured Embryos	No. (%) of Cleaved Embryos	No. (%) of Embryos Developed	
			8–16 Cell	Blastocyst
Control	386	303 (78.3 ± 0.5) ^b^	226 (58.7 ± 0.8) ^b^	120 (31.1 ± 0.5) ^b^
D-gal	418	276 (63.8 ± 2.6) ^a^	186 (43.6 ± 1.5) ^a^	76 (17.9 ± 1.0) ^a^
TERT	394	318 (78.9 ± 0.8) ^b^	262 (65.3 ± 2.2) ^c^	158 (39.1 ± 0.8) ^c^

Different superscripts in the same column indicate significant differences (*p* < 0.05). Data were obtained from five replicates.

**Table 2:** Table of forward and reverse primers of genes.

Gene	Primer Sequence	Product Length
OCT4	5′-CCA-CCC-TGC-AGC-AAA-TTA-GC-3′	68
	5′-CCA-CAC-TCG-GAC-CAC-GTC-TT-3′	
SOX2	5′-GGT-TGA-CAT-CGT-TGG-TAA-TTT-ATA-ATA-GC-3′	88
	5′-CAC-AGT-AAT-TTC-ATG-TTG-GTT-TTT-CA-3′	
CDX2	5′-GCA-AAG-GAA-AGG-AAA-ATCA-ACA-A-3′	120
	5′-GGC-TCT-GGG-ACG-CTT-CT-3′	
mtTFA	5′-CAA-ATG-ATG-GAA-GTT-GGA-CG-3′	148
	5′-AGC-TTC-CGG-TAT-TGA-GAC-C-3′	
NRF1	5′-CCC-AAA-CTG-AGC-ACA-TGG-C-3′	162
	5′-GTT-AAG-TAT-GTC-TGA-ATC-GTC-3′	
GAPDH	5′-CCC-AGA-ATA-TCA-TCC-CTG-CT-3′	185
	5′-CTG-CTT-CAC-CAC-CTT-CTT-GA-3′	

## Data Availability

Not applicable.

## Abbreviations

TERT	Telomerase reverse transcriptase
COCs	Cumulus oocyte complexes
OPU	Ovum pick-up Carnitine
FGFR1	Fibroblast growth factor receptor 1
CAG	Cycloastragenol
KLB	β-Klotho
MEF	Maturation promoting factor
MAPK	Mitogen-activated protein kinase
MEF	Maturation promoting factor
ROS	Reactive Oxygen Species
PN	pronuclear
BL	Blastocyst
SF-1	Steroidogenic factor-1
sKL	secreted Klotho
GV	Germinal vesicle
